# Inflammation-Based Prognostication in Advanced-Stage NSCLC: A Retrospective Cohort Study

**DOI:** 10.3390/cancers17172910

**Published:** 2025-09-05

**Authors:** Carina Golban, Cristina-Miriam Blaga, Norberth-Istvan Varga, Alina Gabriela Negru, Delia Hutanu, Sorin Saftescu, Serban Mircea Negru

**Affiliations:** 1 Doctoral School, Department of General Medicine, “Victor Babes” University of Medicine and Pharmacy, Eftimie Murgu Square No. 2, 300041 Timisoara, Romania; carina.golban@umft.ro (C.G.); cristina.ciortuz@umft.ro (C.-M.B.); 2 Department of Cardiology, “Victor Babeș” University of Medicine and Pharmacy, Eftimie Murgu Square No. 2, 300041 Timisoara, Romania; alinanegru@umft.ro; 3 Department Biology-Chemistry, Faculty of Chemistry-Biology-Geography, West University of Timisoara, Pestalozzi, 16, 300315 Timisoara, Romania; delia.hutanu@e-uvt.ro; 4 Department of Oncology, Faculty of Medicine, Victor Babeş University of Medicine and Pharmacy Timisoara, Eftimie Murgu Square 2, 300041 Timisoara, Romania; sorin.saftescu@umft.ro (S.S.); serban.negru@umft.ro (S.M.N.)

**Keywords:** lung cancer, non-small cell lung cancer, NSCLC, neutrophil-to-lymphocyte ratio, NLR, progression-free survival, NLR prognostic value

## Abstract

**Background/Objectives**: Neutrophil-to-lymphocyte ratio (NLR), a marker of systemic inflammation, has prognostic value in non-small cell lung cancer (NSCLC), but its longitudinal performance in routine care is unclear. We evaluated baseline and 12-month changes in NLR and hemoglobin in a single-center, Eastern European cohort. **Methods**: In this retrospective study, 180 adults with histologically confirmed NSCLC, diagnosed May 2022–April 2024 at a Romanian tertiary center, were followed until 30 April 2025. Baseline demographics, tumor characteristics, molecular profiles, laboratory parameters, and treatments were extracted from electronic health records. Progression-free survival (PFS) was the primary endpoint, overall survival (OS) the secondary, analyzed using Kaplan–Meier curves and Cox proportional hazards models. An additional treatment-start-anchored sensitivity analysis in treated patients was conducted. **Results**: The cohort (median age 67.8 years, 68.9% stage IV) received chemo-immunotherapy (58.9%), immunotherapy (26.7%), chemotherapy (9.4%), or supportive care (5.0%). Median for PFS was 8.2 months and for OS 14.5 months. A high baseline NLR (≥3, 58.9%) increased progression risk (HR 1.60, 95% CI 1.10–2.32, *p* = 0.014), with a trend for worse OS (HR 1.45, 95% CI 0.99–2.12). A 12-month NLR increase (62.2%) further elevated progression risk (HR 1.52, 95% CI 1.05–2.20, *p* = 0.026). Low hemoglobin (<12 g/dL) had a non-significant effect (HR 1.38, 95% CI 0.97–1.96, *p* = 0.074). PD-L1 ≥ 50% and chemo-immunotherapy correlated with longer PFS. Findings were consistent in the treatment-start anchored sensitivity analysis. **Conclusions**: These exploratory findings suggest that inexpensive hematologic markers can complement clinical assessment in advanced-stage NSCLC; prospective multi-center validation is warranted.

## Simple Summary

Simple, routine blood tests could potentially reveal insightful information about the severity and prognosis of patients with non-small cell lung cancer. We studied two simple measures: (1) the balance between neutrophils and lymphocytes, which reflects systemic inflammation, and (2) hemoglobin, which measures anemia. We wanted to check whether these two parameters are associated with our two main endpoints: progression-free survival and overall survival. Among 180 adult patients, a higher white-cell balance at diagnosis and a rise over the first year were linked to earlier cancer worsening. Lower hemoglobin showed a weaker, non-significant link to earlier worsening. Patients treated with combined chemotherapy and immunotherapy tended to do better than with either alone. Because these tests were inexpensive and already collected in practice, they could help doctors identify higher-risk patients and tailor follow-up and treatment. However, larger, multicentric studies are needed to confirm our findings.

## 1. Introduction

Non-small cell lung cancer (NSCLC) represents the predominant subtype of lung cancer, accounting for approximately 85% of all lung cancer diagnoses, in contrast to small cell lung cancer (SCLC), which comprises the remaining cases [1,2]. Globally, lung cancer remains the leading cause of cancer-related mortality, with an estimated 2.2 million new cases and 1.8 million deaths annually as of 2022 [2,3,4,5]. In Romania, lung cancer incidence mirrors global trends, with smoking as the primary risk factor, alongside environmental exposures such as radon and air pollution, contributing to its prevalence [5,6,7,8]. NSCLC is classified using the eighth edition of the TNM staging system, where advanced stages, particularly stage IV, are associated with a poor prognosis, with 5-year survival rates often below 10% [9,10]. This high burden underscores the need for effective prognostic tools to guide clinical management and improve patient outcomes.

Prognostic factors in NSCLC play a critical role in risk stratification, treatment planning, and predicting survival outcomes [11,12]. Among these, hematological parameters such as the neutrophil-to-lymphocyte ratio (NLR) and hemoglobin levels have emerged as accessible and cost-effective biomarkers [13,14,15,16]. NLR, a marker of systemic inflammation, reflects the balance between pro-tumor neutrophilic activity and anti-tumor lymphocytic response, with higher values associated with worse survival in NSCLC [13,14,15]. Similarly, low hemoglobin levels, indicative of anemia and tumor hypoxia, have been linked to reduced progression-free survival (PFS) and overall survival (OS), particularly in advanced stages [16,17]. Additional biomarkers, such as the PD-L1 tumor proportion score and molecular alterations including EGFR and ALK mutations, also influence prognosis and guide targeted therapies in NSCLC [18,19,20,21,22,23,24]. These biomarkers are routinely measured in clinical practice, offering a practical means to assess prognosis without additional cost or invasive procedures.

While the prognostic value of the NLR and hemoglobin has been established, further research is needed to fully understand their dynamic changes over time and their impact in real-world settings. Many prior studies have focused on baseline measurements, often within controlled clinical trials, which may not fully capture the heterogeneity of routine clinical practice [25,26,27,28,29]. For instance, a multicenter real-world study by Matsumoto et al. showed that both a baseline NLR of ≥5 and an early on-treatment rise in the NLR independently predicted shorter progression-free and overall survival in advanced NSCLC patients receiving first-line chemo-immunotherapy, yet longitudinal NLR trajectories remain underexplored [25]. Additionally, real-world studies have sometimes been limited by smaller cohort sizes or less comprehensive subgroup analyses, such as by stage, PD-L1 expression, or treatment type. Additional studies in a high-volume tertiary center setting would be beneficial to evaluate both baseline and dynamic hematological parameters, capturing their prognostic impact across diverse patient profiles.

This study aims to evaluate the prognostic impact of baseline and dynamic NLRs and hemoglobin levels on survival outcomes in NSCLC patients. We sought to assess their association with progression-free survival (PFS) as the primary endpoint and overall survival (OS) as the secondary endpoint in a retrospective cohort study. Additionally, we examined treatment response according to RECIST 1.1 criteria, which standardizes tumor response assessment in solid tumors, and conducted subgroup analyses by stage, PD-L1 tumor proportion score, treatment type, and molecular markers to provide a comprehensive understanding of these biomarkers’ utility in real-world clinical practice.

## 2. Materials and Methods

### 2.1. Study Design and Population

This retrospective observational cohort study was conducted at the “Oncohelp” Hospital of Timisoara, a major tertiary referral center, serving a population of more than 2 million residents from the western and south-western regions of Romania. Patients with histologically confirmed non-small cell lung cancer (NSCLC-adenocarcinoma, squamous-cell or large-cell carcinoma) diagnosed between 1 May 2022, and 30 April 2024, were identified through the hospital electronic health record (EHR). Tumors were staged according to the 8th edition of the TNM classification [10]. Each patient was followed from the date of diagnosis until radiological or clinical progression (assessed with RECIST v1.1), death, or the study cutoff date of 30 April 2025, resulting in a variable follow-up period of approximately 12–36 months. In addition to the diagnosis-anchored primary analysis, we conducted a sensitivity analysis re-anchoring survival time at treatment initiation among patients who received systemic therapy. The median follow-up was estimated using the reverse Kaplan–Meier method, and survival analyses employed restricted mean survival time (RMST) at 12 months to account for unequal risk periods due to variable entry times. The study period was selected to maximize sample size and event capture within the constraints of available electronic health records (EHRs) and ethical approval. Progression-free survival (PFS) was chosen as the primary endpoint and overall survival (OS) as the secondary endpoint of this study.

The following inclusion criteria were used for cohort inclusion: (1) age ≥ 18 years; (2) histologically confirmed NSCLC (adenocarcinoma, squamous-cell carcinoma, large-cell carcinoma); (3) diagnosis within the accrual window, as per our ethics approval; (4) available baseline hematological parameters (neutrophils, lymphocytes, hemoglobin); and (5) at least one follow-up assessment documented in the EHR. Patients with small cell or other non-NSCLC histologies or with missing baseline laboratory or follow-up data were excluded.

To address treatment-by-indication bias, inverse probability of treatment weighting (IPTW) was planned for objective response rate (ORR) comparisons, adjusting for stage and PD-L1 Tumor Proportion Score (TPS). The median follow-up was estimated with the reverse Kaplan–Meier method, and survival analyses employed restricted mean survival time (RMST) truncated at 12 months, a horizon that included 85% of all progression or death events in this cohort and was therefore common to every patient regardless of entry date.

### 2.2. Data Collection

Data were extracted from the hospitals’ EHR system, encompassing medical records, laboratory reports, pathology results, and radiology assessments. Data collection occurred between 1 May 2022, and 30 April 2025, to ensure all follow-up data up to the study cutoff (30 April 2025) were included. Variables were collected at baseline (within 7 days of diagnosis) and at follow-up intervals (approximately at 6, 12, 24, and 36 months, or earlier if clinically indicated), reflecting routine clinical check-ups.

Demographic and clinical data encompassed age, sex, smoking status (never, former, or current, including pack-years), Eastern Cooperative Oncology Group (ECOG) performance status (0–4), and comorbidities, coded using the Charlson comorbidity index (CCI). Hematological parameters, including neutrophils (×10^9^/L), lymphocytes (×10^9^/L), hemoglobin (g/dL), platelets (×10^9^/L), and the calculated neutrophils-to-lymphocytes ratio (NLR), were recorded at baseline and at each follow-up interval to assess dynamic changes. A NLR increase was defined as any positive change in the NLR (ΔNLR > 0) compared with baseline. Biological data included PD-L1 tumor proportion score (<1%, 1–49%, ≥50%), molecular markers (EGFR, ALK, ROS1, KRAS, BRAF status), lactate dehydrogenase (U/L), and albumin (g/L), collected at baseline. Histopathological data comprised histology (adenocarcinoma, squamous, or large-cell carcinoma), TNM staging (T, N, M components), overall stage (I–IV), and thyroid transcription factor-1 expression, recorded at baseline.

Tumor histologic subtypes (e.g., adenocarcinoma, squamous, etc.) and actionable driver mutations (e.g., EGFR, ALK, KRAS) were abstracted from pathological reports. Because these subgroups were small (e.g., 18% adenocarcinoma with EGFR/ALK alterations), we did not formally test interactions between histologic/molecular subtypes and NLR or hemoglobin. During the study period, comprehensive molecular profiling was performed according to international guidelines, which recommend testing all adenocarcinomas and other non-squamous NSCLC and selectively testing squamous-cell carcinomas when clinical factors (e.g., never/light smoking, young age or small biopsy) suggest a targetable driver.

Imaging data, including tumor size (cm, from computed tomography) and number and location of metastatic sites (e.g., brain, liver, lung), were collected at baseline, with tumor response assessed per Response Evaluation Criteria in Solid Tumors (RECIST 1.1): complete response (CR), partial response (PR), stable disease (SD), or progressive disease (PD) at regular check-ups (6-, 12-, 24-, and 36-month intervals) based on routine CT scans. Treatment data, including type (chemotherapy, immunotherapy, or combination), start date, line of therapy (first, second), number of cycles completed, and end date, were recorded at baseline and updated during follow-up.

Outcome data included (1) progression-free survival (PFS), defined as the time from the index date to documented progression or death from any cause; and (2) overall survival (OS), defined as the time from the index date to death from any cause. For the primary analysis, the index date was the date of histologic diagnosis (available for all patients). We additionally performed a sensitivity analysis among patients who received systemic therapy, re-anchoring the index date at the start of first-line systemic therapy (chemotherapy, immunotherapy, or chemo-immunotherapy). For patients managed with supportive care alone, OS was measured from diagnosis. For treated patients we also recorded the diagnosis-to-treatment interval (days) for use in adjusted models.

Key variables (e.g., stage, NLR, RECIST) were validated through double abstraction by two independent researchers, with random audits of 10% of records yielding an inter-rater reliability of Cohen’s kappa 0.85–0.90.

### 2.3. Ethical Considerations

The study followed the principles of the Helsinki Declaration on medical Protocol and Ethics and was approved by the Ethics Committee of the Oncohelp Hospital of Timisoara (approval number 187/04.11.2024). Upon hospital admission, patients provided general consent for data use, as per general clinical practice. As a retrospective study using existing medical records, individual patient consent was waived by the Ethics Committee. All data were anonymized in compliance with the General Data Protection Regulation (GDPR) and Romanian national regulations and were de-identified for analysis. Only research members involved in data collection (C.G., S.S., and S.M.N.) had access to patients’ full names, while others worked with initials to ensure confidentiality.

### 2.4. Statistical Analysis

Statistical analyses were performed using SPSS version 26 (IBM, Armonk, NY, USA). Continuous variables were assessed for normality of distribution using the Shapiro–Wilk test, with non-normal distributions reported as medians (and interquartile ranges). Categorical variables were reported as frequencies and percentages. Baseline characteristics were compared across groups using the Mann–Whitney U test for continuous variables, and the chi-square test for categorical variables. The median follow-up durations were estimated using the reverse Kaplan–Meier method to account for censoring. Progression-free survival (PFS, primary endpoint) and overall survival (OS, secondary endpoint) were analyzed using Kaplan–Meier curves, with comparisons between groups via the log-rank test. Hazard ratios (HRs) and 95% confidence intervals (CIs) were estimated using Cox proportional hazards models. A sensitivity analysis repeated the RMST calculation at 24 months among patients whose potential follow-up was ≥24 months to confirm robustness of the results. Objective response rate (ORR; complete or partial response per RECIST 1.1) was compared across treatment types, PD-L1 TPS, and hematological parameters using chi-square tests, with inverse probability of treatment weighting (IPTW) applied to adjust for treatment-by-indication bias. Logistic regression models were used to assess associations between the ORR and baseline NLR or hemoglobin. Dynamic changes in the NLR and hemoglobin were compared across groups using the Mann–Whitney U test. The primary endpoint (overall and by stage) was prespecified; all subgroup comparisons (e.g., PD-L1 categories, treatment type, ECOG strata, molecular subgroups) were exploratory. We therefore report unadjusted (“nominal”) *p*-values for subgroup tests and emphasize effect sizes with 95% CIs. Results of subgroup analyses should be interpreted cautiously due to multiplicity; family-wise or FDR adjustments were not applied.

The primary time origin for survival analyses was the date of diagnosis. We repeated all survival analyses with time re-anchored at the start of first-line systemic therapy among treated patients, as a sensitivity analysis. Multivariable Cox models were adjusted for age, sex, stage (I–IV), ECOG performance status, smoking status (ever/never), PD-L1 tumor proportion score (<1%, 1–49%, ≥50%), treatment category (chemotherapy, immunotherapy, chemo-immunotherapy), and line of therapy (first vs. ≥second). In models restricted to treated patients, we additionally adjusted for the diagnosis-to-treatment interval (days). Proportional hazards were evaluated via Schoenfeld residuals; no material violations were detected. RMST was estimated at 12 months for both time origins, with a 24-month RMST sensitivity among patients with a potential follow-up ≥24 months.

## 3. Results

### 3.1. Overview of Study Population

After applying the inclusion and exclusion criteria, a final cohort consisting of 180 patients remained. They had a histologically confirmed diagnosis of non-small cell lung cancer (NSCLC) between 1 May 2022 and 30 April 2024 and were treated and followed up at the Oncohelp Hospital of Timisoara, Romania. Their key baseline characteristics are presented in Table 1.

The cohort had a median age of 67.8 years (IQR 62–74), with 62.8% male patients, and was predominantly composed of advanced-stage cases, reflecting the referral center’s complex case load. Adenocarcinoma was the most common histology (56.7%), followed by squamous-cell carcinoma (28.3%) and large-cell carcinoma (15.0%), with 68.9% of patients at stage IV, 21.7% at stage III, and 9.4% at stages I–II. Most patients (76.6%) were ever-smokers, with a median Charlson comorbidity index (CCI) of 2 (IQR 1–3), and the median Eastern Cooperative Oncology Group (ECOG) performance status was 1 (IQR 0–2), indicating a functional population despite comorbidities such as hypertension and chronic obstructive pulmonary disease.

The baseline tumor size had a median of 4.2 cm (IQR 2.8–5.6 cm), with 68.9% (*n* = 124) of patients having metastatic disease (median one site, IQR 0–2). PD-L1 expression was distributed as <1% in 31.7%, 1–49% in 38.3%, and ≥50% in 30.0%, with thyroid transcription factor-1 (TTF-1) positive in 69.2% of adenocarcinomas.

In our cohort, 93 of 102 adenocarcinoma cases (91.2%) underwent molecular testing, compared with 20 of 51 squamous-cell carcinomas (39.2%) and 9 of 27 large-cell carcinomas (33.3%). Among those tested (see Table 1 for denominators), EGFR mutations were identified in 26 cases, ALK rearrangements in 10 cases, and KRAS mutations in 44 cases. The median baseline lactate dehydrogenase (LDH) was 248 U/L (IQR 209–344 U/L), and albumin was 34.8 g/L (IQR 29.6–39.1 g/L). Over the follow-up period, 63.3% of patients experienced progression or death, aligning with expected event rates for a stage IV-heavy cohort.

Treatment regimens included chemo-immunotherapy combinations (58.9%, *n* = 106; e.g., pembrolizumab with carboplatin and pemetrexed, primarily in stage IV), immunotherapy alone (26.7%, *n* = 48; e.g., pembrolizumab or atezolizumab, often in PD-L1 ≥ 1%), and chemotherapy (9.4%, *n* = 17) or supportive care (5.0%, *n* = 9) in early-stage or palliative settings. For ALK rearrangement-positive patients, treatment consisted of ALK inhibitors, with alectinib as the first-line therapy and lorlatinib upon progression.

With 180 patients and 114 progression/death events (63.3%), a post hoc power calculation using the Schoenfeld method (α = 0.05, two-sided) confirmed that the study achieved 80% power to detect a hazard ratio of ≥1.50 for a high (NLR ≥ 3) versus low NLR on progression-free survival (PFS), based on the observed 12-month PFS of 40% in the low-NLR group and the observed event rate.

### 3.2. Baseline and Longitudinal Hematological Parameters

At baseline, the cohort of 180 NSCLC patients had a median neutrophil-to-lymphocyte ratio (NLR) of 3.6 (IQR 2.5–5.2), with 58.9% (*n* = 106) exhibiting a high NLR (≥3). The median hemoglobin level was 11.7 g/dL (IQR 10.2–13.0), with 43.3% (*n* = 78) having low hemoglobin (<12 g/dL). A high NLR was significantly associated with stage IV disease (64.2% of stage IV patients vs. 46.4% of stages I–III, χ^2^ = 6.82, *p* = 0.009) and a ECOG performance status of 2–3 (68.6% vs. 53.5% for ECOG 0–1, χ^2^ = 4.15, *p* = 0.042). Low hemoglobin was more frequent in stage IV patients (48.4% vs. 32.1% for I–III, χ^2^ = 4.89, *p* = 0.027) and ever-smokers (46.9% vs. 34.6% for never-smokers, χ^2^ = 3.12, *p* = 0.077), though the latter association was not statistically significant. Baseline NLR and hemoglobin levels did not differ significantly by sex or histology (*p* = 0.131, Mann–Whitney U test).

By histology, the median baseline NLR (IQR) was 3.5 (2.4–5.1) in adenocarcinoma, 3.7 (2.6–5.5) in squamous-cell carcinoma, and 3.8 (2.7–5.6) in large-cell carcinoma; these differences were not statistically significant (*p* = 0.198). The proportion of patients with a high NLR (≥3) was 60/102 (58.8%), 31/51 (60.8%) and 15/27 (55.6%) in adenocarcinoma, squamous-cell and large-cell carcinoma, respectively.

Over the follow-up period, the NLR increased, with a median change of +0.7 (IQR −0.5 to +2.1) by 12 months, observed in 62.2% of patients (*n* = 112), particularly those receiving chemo-immunotherapy (67.0% vs. 54.2% for immunotherapy alone, χ^2^ = 3.88, *p* = 0.049). Hemoglobin decreased slightly, with a median change of −0.6 g/dL (IQR −1.4 to +0.8) by 12 months, affecting 53.9% of patients (*n* = 97), more pronounced in stage IV (58.1% vs. 42.9% for I–III, χ^2^ = 4.22, *p* = 0.040). Dynamic changes in the NLR were significantly higher in patients with an ECOG performance status of 2–3 (median ΔNLR +1.1, IQR 0.3–2.4 vs. +0.5, IQR −0.2 to +1.8 for ECOG 0–1, U = 2784, *p* = 0.032, Mann–Whitney U test). The 36-month follow-up data were excluded due to the limited number of patients (*n* = 38) with available measurements, resulting from high progression and mortality rates, particularly among stage IV patients. Table 2 summarizes the baseline and follow-up hematological parameters.

### 3.3. Treatment Patterns and Tumor Response

Among the 180 NSCLC patients, 106 (58.9%) received chemo-immunotherapy combinations (median cycles, IQR 3–7; e.g., carboplatin, pemetrexed, pembrolizumab), 48 (26.7%) received immunotherapy alone (median 10 cycles, IQR 6–14; e.g., pembrolizumab, atezolizumab), and 26 (14.4%) received chemotherapy (median 4 cycles, IQR 2–6; e.g., docetaxel) or supportive care. First-line therapy was administered in 153 patients (85.0%), second-line in 18 (10.0%), and supportive care in 9 (5.0%), reflecting the predominantly advanced-stage cohort (68.9% stage IV). Treatment distribution did not differ significantly by sex or histology (*p* > 0.05, chi-square test), but stage IV patients were more likely to receive chemo-immunotherapy (64.5% vs. 46.4% for stages I–III, χ^2^ = 5.12, *p* = 0.024). Objective response rate (ORR) comparisons were adjusted using inverse probability of treatment weighting (IPTW) to account for treatment-by-indication bias, with details to be provided in the statistical analysis section.

At 6 months, the tumor response per RECIST 1.1 criteria showed an ORR (complete response [CR] or partial response [PR]) of 23.3% (*n* = 42; 2.2% CR, *n* = 4; 21.1% PR, *n* = 38) and a disease control rate (DCR; CR, PR, or stable disease [SD]) of 57.8% (*n* = 104, including 34.5% SD, *n* = 62), with 42.2% (*n* = 76) experiencing progressive disease (PD). By 12 months, the ORR decreased to 16.7% (*n* = 30) and DCR to 42.2% (*n* = 76), with PD rising to 53.3% (*n* = 96). Higher PD-L1 TPS (≥50%) was associated with an increased ORR (34.4% vs. 14.0% for <1%, χ^2^ = 8.76, *p* = 0.003), particularly in immunotherapy-treated patients. A high baseline NLR (≥3, 58.9%) correlated with a lower DCR (51.9% vs. 66.2% for NLR < 3, χ^2^ = 4.33, *p* = 0.037), and low hemoglobin (<12 g/dL, 43.3%) was linked to higher PD (48.7% vs. 37.3% for ≥12 g/dL, χ^2^ = 4.01, *p* = 0.045). Stage IV patients had a lower ORR (19.4% vs. 33.9% for stages I–III, χ^2^ = 6.24, *p* = 0.013), and chemo-immunotherapy yielded a higher ORR than immunotherapy alone (27.4% vs. 16.7%, χ^2^ = 4.08, *p* = 0.043). The tumor response outcomes are summarized in Table 3.

### 3.4. Survival Outcomes

**Figure 1:**
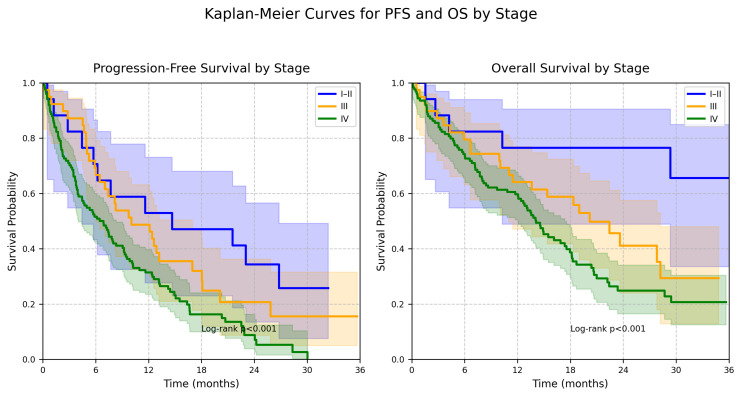
Kaplan–Meier curves for progression-free and overall survival by stage in NSCLC.

The median progression-free survival (PFS) for the overall cohort (all 180 patients) was 8.2 months (IQR 4.1–14.7), with a restricted mean survival time (RMST) of 6.8 months at 12 months. By stage, the median PFS was 7.0 months (IQR 3.8–12.2) for stage IV (*n* = 124), 12.5 months (IQR 6.9–18.3) for stage III (*n* = 39), and 20.8 months (IQR 12.4–28.6) for stages I–II (*n* = 17), with significant differences (log-rank *p* < 0.001). The median overall survival (OS) was 14.5 months (IQR 7.6–24.9), with a RMST of 9.5 months at 12 months. OS by stage was 12.0 months (IQR 6.5–19.8) for stage IV, 24.2 months (IQR 13.7-not reached) for stage III, and 35.9 months (IQR 22.8-not reached) for stages I–II (log-rank *p* < 0.001). Kaplan–Meier curves for PFS and OS are presented in Figure 1.

A high baseline neutrophil-to-lymphocyte ratio (NLR ≥ 3, 58.9%) was associated with worse survival, with an adjusted hazard ratio (HR) of 1.60 (95% CI 1.10–2.32, *p* = 0.014) for PFS and 1.45 (95% CI 0.99–2.12, *p* = 0.056) for OS, after adjusting for age, sex, stage, ECOG, smoking status, PD-L1 TPS, and treatment-start delay. Low baseline hemoglobin (<12 g/dL, 43.3%) was linked to poorer PFS (HR 1.38, 95% CI 0.97–1.96, *p* = 0.074) and OS (HR 1.28, 95% CI 0.88–1.86, *p* = 0.193). Dynamic changes at 12 months showed that an NLR increase (median ΔNLR +0.7, 62.2% of patients) significantly predicted worse PFS (HR 1.52, 95% CI 1.05–2.20, *p* = 0.026) but not OS (HR 1.35, 95% CI 0.92–1.98, *p* = 0.124). A hemoglobin decrease (median Δhemoglobin −0.6 g/dL, 53.9%) had a modest effect on PFS (HR 1.22, 95% CI 0.85–1.75, *p* = 0.279) and OS (HR 1.15, 95% CI 0.79–1.67, *p* = 0.462).

#### 3.4.1. Sensitivity Analysis with Treatment Initiation as Time Zero

**Figure 2:**
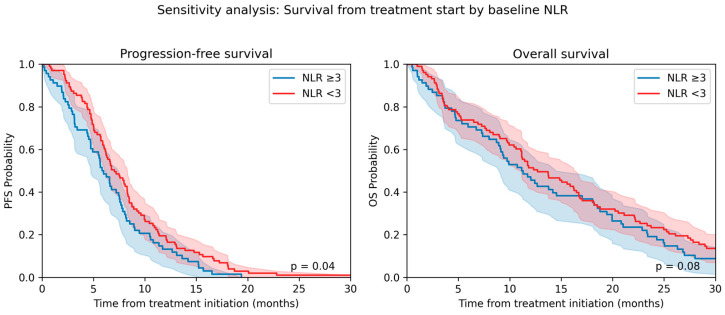
Kaplan–Meier curves for PFS and OS anchored at treatment start (treated subset).

Among patients who received systemic therapy (*n* = 171), re-anchoring time zero to treatment start, rather than at diagnosis, yielded survival estimates comparable to the primary analysis, although slightly shorter: the median PFS fell from 8.2 months in the primary analysis to 6.7 months (IQR 3.2–12.3), and the median OS fell from 14.5 to 12.7 months (IQR 6.8–21.3). Patients with a high baseline NLR (≥3) still had worse outcomes: a median PFS of 5.9 months versus 7.3 months for NLR of < 3 and median OS of 11.1 months versus 12.7 months. Adjusted Cox models anchored at treatment start yielded hazard ratios similar to the primary analysis, with a high NLR associated with a 55% higher risk of progression (HR 1.55, 95% CI 1.05–2.28; *p* = 0.032) and a 40% higher risk of death (HR 1.42, 95% CI 0.93–2.17). A 12-month increase in the NLR continued to predict worse PFS (HR 1.41, 95% CI 1.00–2.05; *p* = 0.049) but not OS. These results suggest that anchoring survival at treatment initiation produces slightly shorter absolute survival estimates yet preserves the relative prognostic importance of baseline and dynamic NLR values. Kaplan–Meier curves are shown in Figure 2.

These numbers represent the treated subset only; supportive-care patients (*n* = 9) do not have a treatment-start anchor.

#### 3.4.2. Exploratory Subgroup Analyses

Exploratory subgroup analyses revealed significant survival differences. Patients with a PD-L1 TPS of ≥50% (*n* = 54) had longer PFS (median 9.8 months, IQR 5.2–16.1) and OS (17.0 months, IQR 9.1–28.4) compared with those with a TPS of <1 % (*n* = 57; PFS 6.5 months, IQR 3.4–11.8; OS 11.2 months, IQR 5.9–19.3; nominal log-rank *p* = 0.003). Chemo-immunotherapy (*n* = 106) was associated with better PFS (median 8.9 months, IQR 4.7–15.3) than immunotherapy alone (*n* = 48; 7.2 months, IQR 3.9–12.8; nominal log-rank *p* = 0.031), with no significant OS difference (*p* = 0.142).

Among the 180 evaluable patients, those with a baseline ECOG performance status of 0–1 (*n* = 129, 71.7%) achieved a median progression-free survival (PFS) of 9.2 months (IQR 5.2–16.1) and a 12-month PFS restricted mean survival time (RMST) of 6.8 months. Their median overall survival (OS) was 17.4 months (IQR 10.3–28.5) with a 12-month OS-RMST of 12.5 months. In contrast, patients with an ECOG performance status of 2–3 (*n* = 51, 28.3%) had a median PFS of 5.1 months (IQR 2.7–9.1) and a 12-month PFS-RMST of 4.1 months, while median OS and 12-month OS-RMST were 9.8 months (IQR 5.4–17.0) and 7.3 months, respectively. No additional hypothesis testing was performed because the ECOG grade was already included as a covariate in the multivariable Cox model.

Among stage IV patients without EGFR/ALK alterations (*n* = 88), the PD-L1 TPS remained a significant prognostic factor despite the absence of targeted therapy. Median progression-free survival (PFS) and overall survival (OS) were 6.0 months (IQR 3.2–9.5) and 11.0 months (IQR 5.8–16.5) for a TPS of <1 % (*n* = 28), 7.8 months (IQR 4.1–12.0) and 13.5 months (IQR 7.2–20.0) for a TPS of 1–49% (*n* = 33), and 9.2 months (IQR 4.9–14.0) and 16.0 months (IQR 8.5–23.5) for a TPS of ≥50% (*n* = 27), respectively. Treatment modalities varied by PD-L1 expression: chemo-immunotherapy predominated in a TPS of <1 % (65%), while immunotherapy alone was most common in a TPS of ≥50% (50%).

Regarding the ALK rearrangement-positive subgroup (*n* = 10), descriptive survival outcomes showed a median PFS of 18.9 months (IQR 14.2–24.5) and a median OS of 33.7 months (IQR 27.6 –44.1). The RMST at 12 months was 9.9 months for PFS and 10.7 months for OS. Due to the small sample size, statistical comparisons were not performed.

Patients with EGFR mutations (*n* = 26), all treated with Osimertinib, had longer PFS (median 11.8 months, IQR 6.5–16.4) compared with wild-type (8.5 months, IQR 4.3–15.0; log-rank *p* = 0.032), reflecting the efficacy of targeted therapy. OS was also improved in EGFR-positive patients (median 23.8 months, IQR 14.3–30.5) compared with wild-type (15.0 months, IQR 7.9–25.8; log-rank *p* = 0.048).

Survival outcomes are summarized in Table 4.

## 4. Discussion

### 4.1. Our Findings and Previous Research

This retrospective cohort study aimed to evaluate the prognostic impact of baseline and dynamic neutrophil-to-lymphocyte ratios (NLRs) and hemoglobin levels on survival outcomes in 180 patients with non-small cell lung cancer (NSCLC), diagnosed and treated at the Oncohelp Hospital, Timisoara, Romania. These hematological parameters were selected due to their accessibility, cost-effectiveness, and potential to provide prognostic insights in a real-world setting, where longitudinal data remain underexplored. We sought to assess their association with progression-free survival (PFS) as the primary endpoint and overall survival (OS) as the secondary endpoint, alongside subgroup analyses by stage, PD-L1 expression, and treatment type.

Our findings highlight the significant prognostic value of the NLR and hemoglobin in NSCLC, offering insights that may be accessible to non-oncologist clinicians. We found that patients with a high baseline NLR, meaning a ratio of 3 or greater, which reflects increased inflammation in the body, faced a higher risk of disease progression or death, with a 60% increased risk compared with those with lower NLR values. Similarly, patients with low hemoglobin levels, below 12 g/dL, indicating anemia, also showed a trend toward earlier disease progression. Additionally, stage IV patients, who made up nearly 70% of our cohort, experienced much shorter survival times, with disease progression typically occurring within 7 months and overall survival around 12 months, compared with over 20 months for progression in early-stage patients. Those with higher PD-L1 expressions, a marker on cancer cells, or receiving combined chemotherapy and immunotherapy treatments, had better outcomes, with progression delayed by about 2–3 months compared with others. These results suggest that simple blood tests like NLRs and hemoglobin can help identify NSCLC patients at higher risk of poor outcomes, enabling closer monitoring or tailored treatment strategies.

Our results support the potential utility of a longitudinal NLR and systemic inflammation monitoring NSCLC. In a recent multicenter real-world study of 280 chemo-immunotherapy-treated patients, Matsumoto et al. [25] reported that a baseline NLR of ≥5 and a non-decreasing NLR at six weeks (ΔNLR ≥ 0) independently predicted inferior PFS (HR 1.74, 95% CI 1.32–2.30) and OS (HR 1.86, 95% CI 1.37–2.51) over a median follow-up of 30 months. Patients who entered treatment with both a NLR of <5 and a falling ΔNLR enjoyed a 2-year OS rate of 58.3%, whereas those with a high-and-rising NLR had a rate of only 5.6%. Our cohort reproduced the same qualitative pattern: a baseline NLR of ≥5 and any on-treatment increase were each associated with a shorter RMST at 12 months and with a two-fold higher hazard of progression or death in multivariable models. The consistency across geographically distinct populations and across treatment regimens (first-line chemo-immunotherapy in Matsumoto versus a mixed real-world case-mix of stages III–IV and systemic modalities in the present study) strengthens the case for incorporating longitudinal NLR monitoring into routine clinical algorithms, particularly in centers without ready access to more sophisticated immune profiling.

Of the 180 patients in the cohort, 112 (62.2%) had a recorded NLR at 12 months, while the remaining 68 either progressed, died, or were lost to follow-up before this time point. Patients without a 12-month measurement tended to have a higher baseline NLR (median 4.1 vs. 3.4) and a higher proportion of stage IV disease. As a result, the dynamic NLR findings may underestimate the prognostic impact of a rising NLR, because early progressors were excluded. Similar longitudinal studies note that declines in event rates over time can reflect either natural resolution or survivor bias [30].

While we found that a ≥0 rise in the NLR over 12 months was associated with higher progression risk, our study design cannot determine whether a rising NLR is a true predictor of poor prognosis or simply a surrogate marker of advancing disease. The 12-month NLR measurement may coincide with tumor progression or systemic inflammation rather than precede it. Consequently, the observed association could reflect concurrent disease activity. Studies in other malignancies suggest that early changes in the NLR can be more informative than baseline values; for example, in hepatocellular carcinoma, patients with a continuous rise in the NLR within the first 3 months after transarterial chemoembolization had significantly higher hazards of death and poorer tumor response [31].

The prognostic significance of NLRs and hemoglobin in our study aligns with previous studies in NSCLC. A high baseline NLR (≥3) was associated with a 60% increased risk of progression (HR 1.60, *p* = 0.014), consistent with findings from Rapoport et al., who reported that a NLR of <5 was associated with better OS in nivolumab-treated patients (HR 2.58 for a NLR of ≥5 vs. <5, *p* = 0.001) [32]. Similarly, Nakaya et al. found that a baseline NLR of ≥5 was linked to worse PFS (HR 2.07, *p* = 0.002) and OS (HR 1.78, *p* = 0.02) in patients treated with nivolumab [33]. Although these studies used a higher cutoff of 5, our use of a NLR of ≥3 may reflect a more sensitive threshold in our cohort, capturing patients with moderate inflammation who still experience poorer outcomes. In contrast, Diem et al. reported a non-significant association between a high NLR (≥5) and PFS in nivolumab-treated NSCLC patients (HR 1.34, *p* = 0.08), suggesting that our lower cutoff of ≥3 may be more sensitive for detecting prognostic effects in a real-world cohort [34]. Templeton et al.’s meta-analysis, using a median cutoff of 4, found a HR of 1.46 for time to recurrence (*p* = 0.005), further supporting the NLR’s predictive role [35]. For hemoglobin, our finding of a trend toward poorer PFS with low hemoglobin (<12 g/dL, HR 1.38, *p* = 0.074) aligns with Caro et al.’s review, which identified anemia as a risk factor for reduced survival in cancer patients (relative risk 1.65, *p* < 0.001) [36]. Svaton et al. also reported that normal hemoglobin levels were associated with better OS in advanced NSCLC treated with bevacizumab plus chemotherapy (*p* = 0.02), though specific HRs were not provided [37]. The non-significant association with OS in our study (HR 1.28, *p* = 0.193) may be due to the high proportion of stage IV patients and varied treatment regimens, reflecting real-world complexities.

Survival outcomes in our study reflect established patterns in NSCLC literature, particularly when stratified by stage and biomarkers. Stage IV patients had a median PFS of 7.0 months and OS of 12.0 months, comparable to the control arm in the KEYNOTE-189 trial (PFS 4.9 months, OS 11.3 months) and real-world data from Tajarernmuang et al. (PFS 5.5 months, OS 14.6 months for anti-PD1 therapy) [28,38]. Patients with a PD-L1 TPS of ≥50% exhibited better survival (PFS 9.8 months, OS 17.0 months) than those with a TPS of <1 % (PFS 6.5 months, OS 11.2 months), consistent with Reck et al.’s findings in the KEYNOTE-024 trial, where pembrolizumab led to a median PFS of 10.3 months in PD-L1 ≥ 50% patients [26]. Chemo-immunotherapy’s association with longer PFS (8.9 months vs. 7.2 months for immunotherapy alone) aligns with the experimental arm in KEYNOTE-189 (PFS 8.8 months) [28]. EGFR mutation status significantly influenced outcomes, with EGFR-positive patients (*n* = 26), all treated with Osimertinib, demonstrating longer PFS (11.8 months, IQR 6.5–16.4) compared with wild-type patients (8.5 months, IQR 4.3–15.0; *p* = 0.032). Our cohort’s high proportion of stage IV disease, combined with an elevated NLR, treatment delays, and comorbidities (median CCI of 2), are factors that might explain why our PFS is lower than the FLAURA trial’s 18.9 months [39]. Our finding that a high baseline NLR predicts worse PFS in NSCLC is also consistent with a study by Popovici et al., who reported that an elevated NLR was associated with increased mortality (HR 1.226, *p* = 0.005), suggesting inflammation-based biomarkers are robust prognostic tools across cancer types in real-world settings [40].

A notable strength of our study is the evaluation of dynamic changes in the NLR and hemoglobin, providing a more comprehensive understanding of their prognostic utility over time. The increase in the NLR over 12 months (median ΔNLR +0.7) was associated with a 52% increased risk of progression (HR 1.52, *p* = 0.026), supporting Palomar-Abril et al.’s findings that an increase in the NLR after chemoradiotherapy was linked to worse OS in stage III NSCLC (HR 2.51, *p* = 0.029) [41]. This longitudinal approach, conducted in a real-world setting, addresses gaps in trial-based studies, which focus on baseline measurements. Our cohort’s diversity, including a high proportion of stage IV patients (68.9%) and varied treatment regimens, enhances the applicability of these findings to routine clinical practice, as seen in population-based studies using SEER data [42].

Several other systemic inflammation markers, such as the platelet-to-lymphocyte ratio (PLR), C-reactive protein (CRP), D-dimer, and lactate dehydrogenase (LDH), have been linked to prognosis in lung cancer and other solid tumors. Although our current study did not aim to report these associations, evidence suggests that these markers complement the NLR in prognostic models [43].

### 4.2. Limitations and Future Research Directions

Despite these contributions, our study has several limitations that warrant consideration. An important limitation is the diagnosis-anchored primary time origin, which can introduce lead-time bias, particularly for patients with delayed treatment initiation. We selected this anchor because diagnosis dates were consistently available for the entire cohort. To address this, we added a sensitivity analysis re-anchoring time at the start of first-line systemic therapy among treated patients, which yielded similar conclusions, and in treated-only models we further adjusted for the diagnosis-to-treatment interval.

Additionally, the retrospective, single-center design may limit generalizability, as patient characteristics and treatment practices may differ across institutions. Generalizability is also limited by the large proportion of advanced (stage IV) disease in our cohort, which warrants future larger prospective studies. Therefore, the results should be interpreted as exploratory and hypothesis-generating, requiring confirmation in larger, independent, multicenter cohorts.

We performed multiple subgroup comparisons (e.g., PD-L1 categories, treatment types, ECOG strata). Because these analyses were exploratory, we did not adjust for multiplicity, and some nominal associations may represent false positives. Accordingly, we prioritized effect sizes and CIs and recommend external validation.

The post hoc nature of the power analysis is a limitation, as it was conducted after data collection and may overestimate the study’s power based on observed results. A prospective power calculation would have provided a more robust basis for sample size determination, and future studies should prioritize a priori power analyses to ensure adequate statistical power.

Although we collected histological and molecular data, our study was not powered to explore whether the prognostic values of the NLR or hemoglobin differ by tumor subtype or driver mutations. Prior studies have shown that the NLR may correlate with specific outcomes in certain histologies; for example, in stage IV NSCLC, a high baseline NLR was associated with the development of brain metastases particularly in adenocarcinoma [44], and a dynamic NLR remained prognostic after accounting for EGFR mutation status and PD-L1 expression [43]. Our dataset was too small to test such interactions, so we must acknowledge this as a limitation and recommend that larger cohorts evaluate the NLR and hemoglobin prognostic values across histological and molecular subtypes.

The absence of adverse event data also restricts our ability to assess the safety profile of treatments in relation to the NLR and hemoglobin. The ALK-positive subgroup was small (*n* = 10), so its survival data were presented descriptively without statistical comparisons. Although our cohort comprises 180 patients with 108 death events, giving an events-per-variable ratio of ≥10 in the multivariable model, we recognize that precision remains lower than in large registry studies.

Although baseline blood counts were drawn prior to treatment, we lacked data on acute infections, chronic inflammatory or autoimmune conditions, corticosteroid or immunosuppressant use, and anemia etiology. These factors can elevate neutrophil counts, lower lymphocytes and hemoglobin, and may confound associations.

Our analysis of 12-month changes in the NLR inherently excludes patients who died or progressed before the 12-month visit. Consequently, the subset with available 12-month NLR measurements represents a healthier group, and the prognostic impact of early NLR dynamics may be underestimated. In longitudinal cohorts, declining incidence rates over time may reflect natural resolution or survivor bias, and even short-term lymphocyte data have been shown to be influenced by survivor bias. Future studies should incorporate earlier and more frequent NLR measurements (e.g., at 6 weeks or 3 months) to minimize this bias and to better understand the temporal relationship between NLR changes and clinical outcomes.

Finally, administration of granulocyte-stimulating agents (G-/GM-CSF) was not systematically recorded in our electronic dataset. Transient neutrophilia induced by these agents could therefore have influenced some follow-up complete blood counts, potentially attenuating the true association between NLR dynamics and outcomes. Prospective studies with dedicated capture of supportive medications are warranted to address this source of variability.

Future studies should adopt a prospective, multicenter design, with an a priori power analysis, enabling balanced enrollment across all TNM stages and the statistical precision that large cohorts provide. Survival analysis should begin at treatment initiation, and diagnosis-to-treatment interval should be handled as a covariate. Expanding molecular subgroups (particularly ALK-rearranged and other rare driver mutations) and integrating treatment-specific variables (immunotherapy, chemo-IO, TKIs) into time-varying Cox or causal-inference frameworks will clarify whether inflammation-based markers retain prognostic value within precision-oncology strata.

## 5. Conclusions

This retrospective cohort study suggests that baseline and dynamic neutrophil-to-lymphocyte ratios (NLRs) and hemoglobin levels are valuable prognostic markers in non-small cell lung cancer, with a high NLR (≥3) and low hemoglobin (<12 g/dL) associated with worse progression-free survival. Dynamic increases in the NLR over 12 months further predicted disease progression, highlighting the importance of longitudinal monitoring. Subgroup analyses revealed better survival outcomes for patients with a PD-L1 TPS of ≥50% and those receiving chemo-immunotherapy, particularly in advanced stages. These findings suggest that routine hematological parameters may complement established clinical factors for risk stratification. Future prospective studies should validate these results across diverse populations and explore their integration into personalized NSCLC management strategies.

## Figures and Tables

**Table 1:** Baseline characteristics of the study population. IQR = 25th–75th percentile; NSCLC = Non-Small Cell Lung Cancer; ECOG = Eastern Cooperative Oncology Group; LDH = Lactate Dehydrogenase.

Characteristic	Value
Age, years, median (IQR)	67.8 (62–74)
Sex, *n* (%)	
Male	113 (62.8%)
Female	67 (37.2%)
Histology, *n* (%)	
Adenocarcinoma	102 (56.7%)
Squamous-cell carcinoma	51 (28.3%)
Large-cell carcinoma	27 (15.0%)
Stage, *n* (%)	
I–II	17 (9.4%)
III	39 (21.7%)
IV	124 (68.9%)
Smoking Status, *n* (%)	
Ever-smoker	138 (76.6%)
Never-smoker	42 (23.3%)
Charlson comorbidity index, median (IQR)	2 (1–3)
ECOG performance status, *n* (%)	
0–1	129 (71.7%)
2–3	51 (28.3%)
Tumor size, cm, median (IQR)	4.2 (2.8–5.6)
LDH, U/L, median (IQR)	248 (209–344)
Albumin, g/L, median (IQR)	34.8 (29.6–39.1)
Metastatic sites, *n*(%)	
0	56 (31.1%)
≥1	124 (68.9%)
Treatment, *n* (%) *	
Chemo-immunotherapy	106 (58.9%)
Immunotherapy alone	48 (26.7%)
Chemotherapy/supportive care	26 (14.4%)
PD-L1 TPS, *n* (%)	
<1%	57 (31.7%)
1–49%	69 (38.3%)
≥50%	54 (30.0%)
Molecular markers, *n*/N tested (%) **	
EGFR mutation	26 (14.4%)
ALK rearrangement	10 (5.6%)
KRAS mutation	44 (24.4%)
Events (progression/death), *n* (%)	114 (63.3%)

* Stage I–II patients’ (*n* = 17) R0 resection (lobectomy = 14, segmentectomy = 3); five received adjuvant cisplatin/vinorelbine and one also received adjuvant radiotherapy. ** Denominators reflect the number with available testing for each marker; patients not tested are coded as unknown.

**Table 2:** Baseline and follow-up hematological parameters. NLR = Neutrophil-to-Lymphocyte Ratio.

Parameter	Baseline	6 Months	12 Months	24 Months
NLR, median (IQR)	3.6 (2.5–5.2)	3.8 (2.7–5.5)	4.3 (3.0–6.0)	4.5 (3.2–6.3)
High NLR (≥3), *n* (%)	106 (58.9%)	110 (61.1%)	112 (62.2%)	115 (63.9%)
Hemoglobin, g/dL, median (IQR)	11.7 (10.2–13.0)	11.4 (9.9–12.7)	11.1 (9.6–12.4)	10.9 (9.4–12.2)
Low Hemoglobin (<12 g/dL), *n* (%)	78 (43.3%)	85 (47.2%)	97 (53.9%)	102 (56.7%)

**Table 3:** Tumor response at 6 months by treatment type, PD-L1 TPS, and stage. ORR = Objective Response Rate; CR = Complete Response; PR = Partial Response; SD = Stable Disease; PD = Progressive Disease; DCR = Disease Control Rate.

Characteristic	CR, *n* (%)	PR, *n* (%)	SD, *n* (%)	PD, *n* (%)	ORR (CR + PR), *n* (%)	DCR (CR + PR + SD), *n* (%)
Overall	4 (2.2%)	38 (21.1%)	62 (34.5%)	76 (42.2%)	42 (23.3%)	104 (57.8%)
Treatment type						
Chemo-immunotherapy (*n* = 106)	3 (2.8%)	26 (24.5%)	36 (34.0%)	41 (38.7%)	29 (27.4%)	65 (61.3%)
Immunotherapy alone (*n* = 48)	1 (2.1%)	7 (14.6%)	17 (35.4%)	23 (47.9%)	8 (16.7%)	25 (52.1%)
Chemo/supportive care (*n* = 26)	0 (0.0%)	5 (19.2%)	9 (34.6%)	12 (46.2%)	5 (19.2%)	14 (53.8%)
PD-L1 TPS						
<1% (*n* = 57)	1 (1.8%)	7 (12.3%)	20 (35.1%)	29 (50.9%)	8 (14.0%)	28 (49.1%)
1–49% (*n* = 69)	1 (1.4%)	15 (21.7%)	24 (34.8%)	29 (42.0%)	16 (23.2%)	40 (58.0%)
≥50% (*n* = 54)	2 (3.7%)	16 (29.6%)	18 (33.3%)	18 (33.3%)	18 (34.4%)	36 (66.7%)
Stage						
I–II (*n* = 17)	1 (5.9%)	5 (29.4%)	7 (41.2%)	4 (23.5%)	6 (35.3%)	13 (76.5%)
III (*n* = 39)	1 (2.6%)	11 (28.2%)	14 (35.9%)	13 (33.3%)	12 (30.8%)	26 (66.7%)
IV (*n* = 124)	2 (1.6%)	22 (17.7%)	41 (33.1%)	59 (47.6%)	24 (19.4%)	65 (52.4%)

**Table 4:** Survival outcomes by stage, PD-L1 TPS, treatment, and molecular markers. NR = not reached.

Characteristic	PFS, Median (IQR), Months	OS, Median (IQR), Months	PFS RMST at 12 Months, Months	OS RMST at 12 Months, Months
Overall	8.2 (4.1–14.7)	14.5 (7.6–24.9)	6.8	9.5
Stage				
I–II (*n* = 17)	20.8 (12.4–28.6)	35.9 (22.8-NR)	10.2	11.8
III (*n* = 39)	12.5 (6.9–18.3)	24.2 (13.7-NR)	8.5	11.0
IV (*n* = 124)	7.0 (3.8–12.2)	12.0 (6.5–19.8)	6.2	8.8
PD-L1 TPS				
<1% (*n* = 57)	6.5 (3.4–11.8)	11.2 (5.9–19.3)	5.9	8.3
1–49% (*n* = 69)	8.4 (4.2–14.9)	14.8 (7.8–25.2)	6.9	9.6
≥50% (*n* = 54)	9.8 (5.2–16.1)	17.0 (9.1–28.4)	7.8	10.4
Stage IV without EGFR/ALK alterations				
TPS <1 (*n* = 28)	6.0 (3.2–9.5)	11.0 (5.8–16.5)	5.3	8.2
TPS 1–49% (*n* = 33)	7.8 (4.1–12.0)	13.5 (7.2–20.0)	6.6	9.2
TPS ≥ 50% (*n* = 27)	9.2 (4.9–14.0)	16.0 (8.5–23.5)	7.4	9.9
Treatment				
Chemo-immunotherapy (*n* = 106)	8.9 (4.7–15.3)	15.2 (8.0–26.1)	7.2	9.8
Immunotherapy alone (*n* = 48)	7.2 (3.9–12.8)	13.5 (7.1–23.7)	6.3	9.1
Chemo/supportive care (*n* = 26)	7.8 (4.0–13.6)	13.0 (6.8–22.4)	6.5	8.9
Molecular markers				
EGFR+ (*n* = 26)	11.8 (6.5–16.4)	23.8 (14.3–30.5)	8.8	10.5
EGFR wild-type (*n* = 154)	8.5 (4.3–15.0)	15.0 (7.9–25.8)	7.0	9.7
ALK+ (*n* = 10) *	18.9 (14.2–24.5)	33.7 (27.6 –44.1)	9.9	10.7
ECOG 0—1 (*n* = 129)	9.2 (5.2–16.1)	17.4 (10.3–28.5)	6.8	12.5
ECOG 2—3 (*n* = 51)	5.1 (2.7–9.1)	9.8 (5.4–17.0)	4.1	7.3

* ALK outcomes shown descriptively; statistical testing not performed due to small *n*.

## Data Availability

The data presented in this study are available on request from the corresponding author. The data are not publicly available due to legal and ethical considerations.

## Abbreviations

The following abbreviations are used in this manuscript:

NSCLC	Non-Small Cell Lung Cancer
NLR	Neutrophil-to-Lymphocyte Ratio
CCI	Charleson Comorbidity Index
ECOG	Eastern Cooperative Oncology Group
PFS	Progression-Free Survival
OS	Overall Survival
